# Rationality of Prescriptions by Rational Use of Medicine Consensus Approach in Common Respiratory and Gastrointestinal Infections: An Outpatient Department Based Cross-Sectional Study from India

**DOI:** 10.3390/tropicalmed8020088

**Published:** 2023-01-28

**Authors:** Debjit Chakraborty, Falguni Debnath, Suman Kanungo, Sandip Mukhopadhyay, Nabanita Chakraborty, Rivu Basu, Palash Das, Kalpana Datta, Suman Ganguly, Prithwijit Banerjee, Nilima Kshirsagar, Shanta Dutta

**Affiliations:** 1 ICMR—National Institute of Cholera and Enteric Disease, Kolkata 700010, India; 2 R. G Kar Medical College & Hospital, Kolkata 700004, India; 3 College of Medicine and Sagar Dutta Hospital, Kolkata 700058, India; 4 Medical College & Hospital, Kolkata 700073, India; 5 West Bengal State AIDS Prevention & Control Society, Kolkata 700091, India; 6 Indian Council of Medical Research, New Delhi 110029, India

**Keywords:** prescription pattern, diarrhoea, acute respiratory infection, antibiotics prescription rate, rational use of medicine, antimicrobial resistance

## Abstract

Background: Drug utilisation studies are relevant for the analysis of prescription rationality and are pertinent in today’s context of the increasing burden of antimicrobial resistance. Prescriptions for patients with diarrhoea or Acute Respiratory Infection (ARI) have been analysed in this study to understand the prescription pattern among various categories of prescribers in two tertiary care centers. Methods: This cross-sectional study was conducted from August 2019 to December 2020 in the medicine and pediatrics outpatient departments of two government teaching hospitals in West Bengal, India. A total of 630 prescriptions were evaluated against WHO standards. Prescriptions were assessed by a ‘Rational Use of Medicine Consensus committee’ approach. Results: The Fixed Dose Combination (FDC) was used in half of the patients (51%). Both the generic prescription (23.3%) and adherence to hospital formulary rates (36.5%) were low. The antibiotics prescription rate was high (57%), and it was higher for diarrhoea than ARI. Deviations from the standard treatment guidelines were found in 98.9% of prescriptions. Deviations were commonly found with prescriptions written by the junior doctors (99.6%). Conclusion: Irrational prescribing patterns prevail in tertiary care centers and indicate the necessity of awareness generation and capacity building among prescribers regarding AMR and its unseen consequences.

## 1. Introduction

Rational use of medicine (RUM) is an extremely desirable requirement in the healthcare delivery system. The World Health Organization (WHO) has defined RUM as “patients receive medications appropriate to their clinical needs, in doses that meet their own individual requirements, for an adequate period of time, and at the lowest cost to them and their community” [1]. RUM not only reduces the chance of adverse drug reactions, undesirable drug interactions, mortality, morbidity, and the cost of treatment, but also helps in better utilization of resources, prevents ‘financial toxicity’, antimicrobial overuse, and hence, may prevent antimicrobial resistance (AMR) [1,2]. However, it was noted that, globally, even up to 50% of prescriptions suffer from irrationality [3]. Several factors were noted behind the irrational use of drugs, ranging from poor knowledge to pharmaceutical promotions [4,5]. RUM is strongly promoted by the WHO, and a set of ‘recommended optimums’ were set by the WHO as indicators of rational prescriptions [1].

Evaluation of prescription patterns comprises studies mentioning drug utilization, with the main emphasis on rational use of medicine. The problem of irrational use of drugs, especially antibiotics, is prevalent worldwide [6,7]. The problem is further complicated in developing countries [8]. In developing countries, respiratory illnesses and diarrhoeal diseases remain the main causes of morbidity and mortality, particularly in children, accounting for one in five deaths and resulting in 1.5 million annual fatalities [9]. The majority of diarrhoea cases in children, especially under the age of five, are caused by viruses, whereas both bacterial and viral diarrhoeas are found in adults. Although it has been estimated that antibiotics are required in 5% of diarrhoea cases, the use of antibiotics in practise is rampant [10]. According to the WHO, in developing countries, half of all viral respiratory tract infections and viral diarrhoea were treated with antibiotics. Furthermore, antibiotics were not found to be prescribed in 70% of pneumonia cases where their use was an absolute necessity [11]. However, overuse or underuse of medicines, prescribing wrong or ineffective medicines, polypharmacy practices, use of expensive fixed-dose combination products, and misuse of antibiotics are the common forms of irrational prescription [12,13]. Since both diarrhoea and acute respiratory infections are major public health problems, particularly in children, specific management guidelines were developed by the WHO. Countries such as India came up with their own guidelines to be used in different State Government Health Departments as well [9,14,15]. However, such guidelines are actually tailormade for public health practitioners practicing in low-resource peripheral health settings. In a country such as India, where both the government and private healthcare systems look at the health issues of its 1.3 billion population, achieving RUM remains a daunting task. The primary objectives of this study were to assess prescribing patterns using the WHO criteria for prescription evaluation and to determine the appropriateness of the prescription and the acceptability of the deviations from standard guidelines through a consensus committee approach.

## 2. Materials and Methods

### 2.1. Study Design, Setting

This multicentre cross-sectional study was conducted at two Government teaching hospitals in West Bengal, India from August 2019 to December 2020. The prescriptions were collected from the OPDs of Medicine, Pediatrics, and the Urban Health Training Centre (UHTC) clinic.

### 2.2. Sample Size Calculation

For prescription evaluation studies, according to the WHO, a minimum of 600 prescriptions [16] are required to be studied. Consecutive sampling was conducted to collect the required number of prescriptions. 

### 2.3. Study Enrolment

#### 2.3.1. Inclusion & Exclusion Criteria

We included the prescriptions of patients (adults or children) presenting with symptoms of acute diarrhoea or ARI in the OPD of the study sites and provided written consent to capture and copy the prescription for review. The prescriptions included in the study had details such as the signs and symptoms and/or provisional or final diagnosis of acute diarrhoea/ARI. Prescriptions from critically ill patients or those who did not give consent were excluded. 

#### 2.3.2. Data Collection

Prescriptions of the patients attending the study sites were screened for eligibility and enrolled for the study after application of inclusion criteria by capturing a photograph of it. The photographed prescription, demographic details, relevant clinical information, diagnosis, and medication details were abstracted in the case record forms. 

#### 2.3.3. Data Management and Analysis Plan

The data management system is comprised of data entry, cleaning, back-up, and the generation of regular reports. Built-in quality control mechanisms were developed to ensure data quality and confidentiality. Prescriptions were analysed using the mentioned WHO indicators. Information was collected about the prescriber also in terms of their hierarchical designation, e.g., general resident doctors, post-graduate residents, and senior doctors (Medical officer/Demonstrator/Clinical Tutor, Assistant Professor, Associate Professor and Professor).

A house staff is a junior resident who has completed undergraduate medical degree (MBBS) and internship but yet to join for a post graduate course. Faculty in clinical disciplines include Residential Medical Officer (RMO) cum Clinical Tutor, Assistant Professor, Associate Professor, and Professor. All have qualified with post-graduate medical degrees (MD).

All the above proportions were compared across age groups (pediatric and adult) and different categories of prescribers. The prescriptions were assessed using a “Consensus Committee approach”.

#### 2.3.4. Assessment of Prescription through Consensus Committee Approach

A Rational Use of Medicine Consensus Committee was formed, including clinicians and clinical pharmacologists, with the objective of developing the assessment framework for diarrhoea and ARI prescriptions. Both clinicians and pharmacologists evaluated the prescriptions independently for appropriateness and the identification of deviation from the standard treatment guidelines (WHO, Ministry of Health & Family Welfare, Government of India, ICMR, 2019 and Standard Treatment Guideline, Institute of Health & Family Welfare, Govt. of West Bengal, 2011) [9,14,15,17].

Since all the guidelines are mostly targeted at general practitioners in public health settings, they do not cover many additional drugs, such as probiotics, antihistamines, leukotriene receptor antagonists, bronchodialators, mucolytics, etc., which are commonly prescribed in medical college settings. In such cases, both pharmacologist and clinician performed the assessment based on the scientific rationale and their clinical expertise.

Furthermore, the pharmacologist judged the prescriptions as appropriate or inappropriate on the basis of the signs and symptoms prescribed, the adverse effects of drugs, the route of administration, the dose (appropriate as per age and body weight, individualization, and the maximum dose per day mentioned for acute drugs), duration being correct as per documented indication, the possibility of drug interaction, and the prescription of generic names. The clinician judged the prescriptions independently as appropriate or inappropriate according to the above criteria as well as their clinical judgement, particularly optimising symptom remission and tolerating adverse effects. Acceptability of Deviation was determined using the Table 1:

In case of a disagreement between pharmacologist and clinician, acceptability of the prescription was discussed in the RUMC committee and a case-to-case decision was taken based on the understanding of significant harm over benefit.

Statistical Analysis: descriptive statistics were used to analyse and present the data in terms of proportion (percentage), and mean with standard deviation (SD). The percentage of prescriptions adhering to each indicator was calculated overall and in the subgroups of patient age (below and above 18 years) and different types of prescribers. Agreement between clinicians and pharmacologists for the appropriateness of prescriptions was determined by Cohen’s Kappa statistics.

## 3. Results

Out of total collected 630 prescriptions, 37.3%, 47.5%, and 15.2% were from medicine OPDs, Paediatric OPDs, and UHTC OPD. The prescriber pattern obtained showed a majority of prescriptions (42.9%) written by senior doctors, followed by 39.7% written by general resident doctors, and 17.5% written by post-graduate residents. 

ARI was more common among the collected prescriptions, with 511 (81.1%) prescriptions, and 119 (18.9%) prescriptions were for diarrhoea. It was found that the majority of the patients (63.2%—ARI and 64.7%—diarrhoea) were below the 18-year age group. Around 56% and 49.6% of patients suffering from ARI and diarrhoea, respectively, were female. The majority of ARI prescriptions were written by faculties (43.8%), while maximum diarrhoea prescriptions were written by interns and housestaff (42.9%).

Assessments of prescribing patterns were completed utilizing the WHO criteria. Deviation from WHO criteria were notable in one or more criteria in majority prescriptions (Table 2). Some of the major contributors to deviations were “no mention of signs and symptoms” (90%), “antibiotic prescription rate” (57%), “no mention of body weight” (56%) and Use of FDC (51%). 

The antibiotic prescription rate (APR) was observed to be higher for diarrhoea (65.5%) than ARI (55.2%). The multiple antibiotic prescription rate (MPR) was also high in cases of diarrhoea (30%) as compared to ARI (3.9%). Among the diarrhoea cases, antibiotics were prescribed in 64.4% of acute watery diarrhoea and 66.7% in dysentery. Antibiotics of nitroimidazole class followed by quinolones, cephalosporins were mostly prescribed. Fixed Dose Combination of ciprofloxacin, tinidazole/ofloxacin, ornidazole and ofloxacin, metronidazole was also used. The antibiotics most commonly used in ARI were a combination of Amoxycillin and Clavulinic acid (Beta lactamase inhibitor), followed by Azithromycin, Cephalexin, Cefixime, Co-trimoxazole and Amoxycillin alone. 

### 3.1. Appropriateness of the Prescription and Acceptability of the Deviations through a Consensus Committee Approach

**Figure 1:**
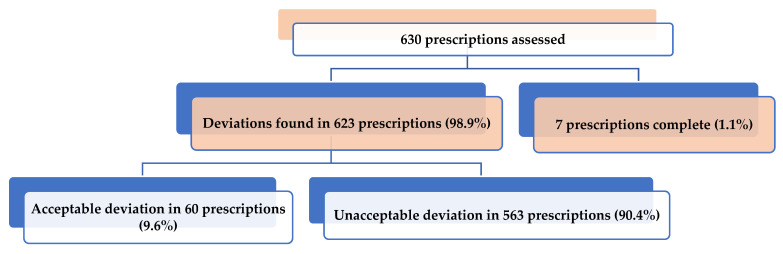
Acceptability of the deviations in assessed prescriptions through a consensus committee approach.

Deviation from the standard guideline as evaluated by the RUMC consensus committee was present in 623 (98.9%) prescriptions (Figure 1). Among them, only 60 (9.5%) were found acceptable. Out of 563 unacceptable deviations, 357 (63.4%) suffered the possibility of Adverse Drug Reactions (ADR), whereas 421 (74.8%) prescriptions had inconsistent or irrational indications. The majority of the unacceptable deviations were due to antibiotics, followed by bronchodilators, antihistaminics, Proton Pump Inhibitor/H 2 receptor blocker/Antacids, Probiotics (Table 3). 

The appropriateness of prescriptions as per clinician and pharmacologist revealed an “agreement between them” in 90.4% of prescriptions (Kappa—0.14) (Table 4). However, only 1.1% of total prescriptions were appropriate according to both clinicians and pharmacologists. There were 9.6% prescriptions that were inappropriate as per a pharmacologist’s recommendation but appropriate as per a clinician’s recommendation. Some common points of disagreement were:

(a) The prescription of antihistaminics in ARI in children has been identified as inappropriate by pharmacologists; however, a 2nd generation antihistaminic (cetirizine) may be considered an acceptable deviation, but a 1st generation (Chlorpheniramine) is unacceptable due to excessive sedation.
(b) The prescription of Azithromycin in URTI was identified as inappropriate by the pharmacologist, as Azithromycin is not a first-line antibiotic, but it was considered an acceptable deviation by the clinician as standard practice.
(c) Drugs prescribed by brand names are considered inappropriate by the pharmacologist, but it was considered an acceptable deviation.
(d) Prescription of albendazole in children or Vitamin D in infants less than 6 months of age, though considered inappropriate by pharmacologists when there is no indication, is considered as acceptable deviation by consensus and adheres to the national program guideline.
(e) ORS prescribed without specific indication is also an acceptable deviation as it causes no apparent harm.

### 3.2. Completeness of Prescriptions—‘Age-Wise’

Body weight was mentioned in 88.5% of prescriptions of patients below 18 years age group. None of the adult prescriptions had body weight mentioned in them. Higher proportion of prescriptions with generic name (27% vs. 17%), and from hospital schedule list (41% vs. 29%) and lower FDC (44% vs. 63%) were observed in prescriptions of <18 years as compared to adults. APR was also lower for children than adults (48% vs. 71%). 

Prescriptions with deviations were slightly lower in children (98.5% vs. 99.5%). However, proportion of acceptable deviations were more in <18 years age group (12% vs. 6%). 

### 3.3. Completeness of Prescriptions across Types of Prescribers

Body weight, signs and symptoms, follow up visit was mentioned most commonly by residents while provisional diagnosis was commonly mentioned by faculties. Prescriptions of all drugs with generic names and from the hospital schedule list were mostly prescribed by residents, while fixed-dose combinations and antibiotics were mostly prescribed by faculties. Deviations were most commonly observed in the prescriptions of junior residents (99.6%), whereas acceptable deviations were more common among the PG residents (15%) (Table 5).

## 4. Discussion

In this study, polypharmacy emerged as a major concern as the average number of drugs prescribed per patient was 4.2 ± 1.9 which is much higher than the WHO standard of ≤2 [18]. However, a few studies have also mentioned a higher average number of drug prescriptions [10,19] per patient, whereas much lower estimates (1.5) were also observed [20]. Several other studies also reported an average range of 2.8–3.2 drugs per patient [21,22,23,24,25]. The higher number of drugs may enhance the chance of adverse drug reactions, antimicrobial resistance, healthcare expenditure and also interfere with prescription adherence. 

Only 23.3% drugs were prescribed by generic names in this study. This is much lower than the standard cut-off of 100% [18]. Higher proportions of generic names were found in studies by Viswanath et al. [26] (62.3%) and Shankar PR et al. [19] (58.1%). Furthermore, in various other studies the proportion of generic names in the prescriptions ranged from 46.2–100% [21,22,27]. The use of generic names is recommended by the government to reduce healthcare costs. It was observed that 53.6% of residents prescribed generic names, compared with only 18% for interns and faculties. However, drugs with Fixed Dose Combinations (FDC) were prescribed in little more than half of the prescriptions and similar findings had been reported by others also [27].

Injectable drugs were not prescribed in any of the prescriptions in this study, as the patients were first-time OPD attendees. The standard value of the proportion of prescriptions where injectables can be prescribed lies between 13.4% and 24.1% [18]. The WHO also recommends lesser use of injectable medications as it increases the cost as well as morbidity and mortality from infections viz. HIV, Hepatitis B and C, air embolism etc. [28].

Overall, 57.0% of prescriptions have at least one antibiotic prescribed. Considering the higher magnitude of infectious diseases in developing countries, WHO has limited the use of antibiotics to <30% of prescriptions for all infectious diseases [18,29]. Consequently, we observed a very high APR and significantly higher in case of diarrhoea and adult patients compared to their counterparts in the study (*p* < 0.05). In India, irrational antibiotic prescription is a serious concern, as reflected by the rates (20% to 72.8%) reported by different studies [21,22,23,24,27]. Antibiotic prescriptions without a provisional diagnosis in a first-time patient support the notion that physicians should cover for immediate medical catastrophes rather than consider backing up antibiotics for future implications in the era of rapidly emerging antimicrobial resistance. We reported a MPR of 10%, which is much lower than studies by Ashraf et al. [30] and Panchal et al. [10]; however, much lower usage of antibiotics of 1 per prescription was reported by Bordoloi et al. [31]. In most of the prescriptions, antibiotics have been prescribed as an empirical therapy without mentioning any provisional diagnosis. A study by Hekster et al. also reported similar findings, where diagnosis was not the deciding factor for prescribing antibiotics in half of the prescriptions [32]. Most episodes of watery diarrhoea in children and sometimes in adults are supposed to be of viral aetiology, where the use of antibiotics is inappropriate; even Acute Respiratory Infection may also be of a viral origin with no indication for antibiotic prescription. This will ultimately contribute to antimicrobial resistance [33,34,35,36]. Also according to ICMR guidelines, antibiotics should not be used for viral respiratory infections and watery diarrhoea and their use should be limited to Streptococcal pharyngitis, bacterial sinusitis and diarrhoea caused due to cholera, amoebiosis, Giarrdiasis, Shigellosis and those caused by Campylobacter or Aeromonas [17]. The Guidelines issued by the State of West Bengal in 2011 also inhibit the inadvertent empirical use of antibiotics [15]. However, our observations are not in accordance with those guidelines.

The most commonly used antibiotic for respiratory infections was a combination of Amoxycillin and Clavulinic Acid, which was corroborated by other studies [37,38]. The most commonly used antibiotics for diarrhoea were Metronidazole alone or with Ciprofloxacin. The easy availability of metronidazole and ciprofloxacin combined with prescriber’s inclination towards a broad spectrum to eliminate the possibility of mixed infection may drive such type of prescriptions.

Deviations from the available treatment guidelines were found in 98.9% of prescriptions, with 90.3% being unacceptable deviations. The unacceptable deviations were in the form of preventable ADR, documentation errors, or drugs prescribed for which rationality could not be explained. A study completed at outpatient clinics in Saudi Arabia reported omissions of various components of the treatment regimen, with some reaching up to 91% incompleteness [39]. Higher adherence to guidelines will actually lead to treatment regimen completion, possibly because of the institutional culture of emphasising the treatment regimen prescription writing [40].

In conclusion, the pattern of prescriptions for diarrhoea and ARI revealed inappropriate practises and non-adherence to the available guidelines (ICMR, state, and WHO). Some common forms of inappropriateness were the use of multiple drugs, the use of brand names, prescribing fixed dose combinations, and the overuse of antibiotics without any rationale.

At the same time, it came to our attention that the available guidelines are more suitable for ‘primary care settings’ where simpler cases are expected to be managed, and probably not the best for managing the ‘complicated’ or ‘referred cases’ in the ‘higher tiers of healthcare’. Hence, healthcare tier specific, evidence-based treatment guidelines may be formulated to minimise the subjective variations in the management approach. 

However, the outcome of irrational prescriptions, such as cure rate, drug-drug interaction, or adverse events, was beyond the scope of the present study. Moreover, diarrhoea and ARI cases are mostly self-limiting and viral in nature. Irrational antimicrobial prescription and consumption not only affect human health adversely but also contribute to environmental contamination with antibiotic residues, Antibiotic Resistant Bacteria (ARBs) and Antibiotic Resistant Genes (ARGs). Judicious use of antibiotics is a prerogative in not only the human health sector but also in animal sectors such as animal husbandry, fisheries, poultry, etc. Unless the problem of Antimicrobial Resistance is tackled through multisectoral involvement, the problem of One Health cannot be fully addressed. In this study, we identified a need for training and education among junior doctors, particularly interns and house staff, regarding rational antibiotic practices, and the findings can be applied to similar practises in other sectors as well. Holistically, in order to establish a One Health approach for the problem of AMR, it requires standardised guidelines, regular capacity strengthening, behaviour change communication, and periodic evaluation at all levels. Our study is a small attempt towards achieving this larger goal. 

## Figures and Tables

**Table 1:** Assessment framework for deviation of prescription through Rational Use of Medicine Consensus Approach.

		Clinician	
		Appropriate	Inappropriate
Pharmacologist	Appropriate	Acceptable/No deviation	Ref to Consensus committee
	Inappropriate	Ref to Consensus committee	Unacceptable

**Table 2:** Completeness of prescriptions as per different criteria according to disease and comparison with WHO core indicators.

Broad Group	Criteria Mentioned in Prescription	Total (*n* = 630)	WHO Core Indicators
		*n* (%)	(%)
Patient and Disease Related Information	Body weight	354 (56.2)	
	Signs and symptoms	569 (90.3)	
	Provisional diagnosis	31 (4.9)	
	Follow up	302 (48.0)	
Drug related information	Mean (SD) no.of drugs prescribed per pescription	4.2 ± 1.9	≤2
	Prescription having all Drugs with generic name	147 (23.3)	100
	Prescription of all Drugs from Hospital schedule list	230 (36.5)	100
	Prescription of all drugs having Fixed Dose Combiation	321 (51.0)	
	Drug formulation not mentioned	4 (0.6)	
	Drug frequency not mentioned	45 (7.2)	
	Drug duration not mentioned	96 (15.3)	
Injectables and antibiotics related information	Prescriptions with injectables	0 (0.0)	13.4–21.1
	Antibiotic Prescription Rate	359 (57.0)	<30

**Table 3:** Distribution of Drug responsible for unacceptable prescription. (*n* = 563 prescriptions, proportions are not mutually exclusive).

Drug Group	Specific Agents Prescribed	No	%
Antibiotics	Amoxycillin, Cefuroxime, Azithromycin, Ofloxacin, Co-trimoxazole.	246	43.7
Bronchodilators	Salbutamol, Terbutaline, Theophylline	240	42.6
H1-Antihistaminics	Chlorpheniramine, Cetrizine, Fexofenadine	128	22.7
Probiotics	Lactobacillus, Bifidobacterium	87	15.4
Proton pump inhibitors	Omeprazone, Pantoprazole, Esomeprazole	70	12.4
Vitamins and mineral supplements	Water soluble vitamins, Iron, Calcium, Zinc.	56	9.9
Leukotrine receptor antagonists	Montelukast	49	8.7
Rehydrating agent	Oral rehydrating salt	44	7.8
H2 receptor blockers	Ranitidine, Famotidine	31	5.5
Non- steroidal anti-inflammatory drugs	Paracetamol, Nimesulide, Diclofenac	28	4.9
Antacids	Magaldrate, Aluminium hydroxide	15	2.6
Anti spasmodic agents	Dicylomine, Drotavarine	15	2.6
Anti-emetic agents	Ondansetron, Domperidone	12	2.1
Corticosteroids	Prednisolone, Deflazacort	7	1.2
Digestive enzymes	Amylase, Lipase	6	1
Nasal decongestants	Oxymetazoline, Xylometazoline	4	0.7
Mucolytic agents	Ambroxol, Guiaphenesin	2	0.3
Non-specific anti-diarrhoeal agent	Racecodotril	1	0.2

**Table 4:** Appropriateness of prescriptions according to clinician and pharmacologist.

Pharmacologist	Clinician		Total	Kappa
	Appropriate	Inappropriate		
Appropriate	7 (1.1)	0 (0.0)	7 (1.1)	0.14
Inappropriate	60 (9.5)	563 (89.4)	623 (98.9)	
Total	67 (10.6)	563 (89.4)	630 (100.0)	

**Table 5:** Completeness of prescriptions as per different criteria across types of prescriber.

Criteria Mentioned in Prescription	Intern & Housestaff (*n* = 250)	Residents (*n* = 110)	Faculty (*n* = 270)
	*n* (%)	*n* (%)	*n* (%)
Body weight	106 (42.4)	89 (80.9)	159 (58.9)
Signs and symptoms	247 (98.8)	109 (99.0)	213 (78.8)
Provisional diagnosis	5 (2.0)	1 (0.9)	25 (9.2)
Follow up	146 (58.4)	92 (83.6)	64 (23.7)
Prescription having all Drugs with generic name	41 (16.4)	59 (53.6)	47 (17.4)
Prescription of all Drugs from Hospital schedule list	81 (32.4)	61 (55.5)	88 (32.6)
Prescription of all drugs having FDC	132 (52.8)	29 (26.4)	160 (59.2)
Prescription with antibiotics	133 (45.2)	29 (26.4)	197 (73.0)
Drug formulation not mentioned	1 (0.4)	1 (0.9)	2 (0.7)
Drug frequency not mentioned	20 (8.0)	3 (2.7)	22 (8.2)
Drug duration not mentioned	45 (18.0)	14 (12.8)	37 (13.7)
Prescription With ORS *	*n* = 51	*n* = 22	*n* = 46
	43 (84.3)	19 (86.3)	35 (76.0)
Prescriptions with deviations	249 (99.6)	106 (96.3)	268 (99.2)
Prescriptions with acceptable deviations **	*n* = 249 11 (4.4)	*n* = 106 16 (15.0)	*n* = 268 33 (12.3)
Prescriptions with chance of ADR ***	*n* = 238 156 (65.5)	*n* = 90 66 (73.3)	*n* = 235 131 (55.7)
Prescriptions with inconsistent/irrational indication ***	*n* = 238 163 (68.4)	*n* = 90 45 (50.0)	*n* = 235 213 (90.6)

* Proportion of prescriptions with ORS has been computed for diarrhoea cases only. ** Proportion of acceptable deviations have been computed out of total deviations in each category. *** Proportion of prescriptions with chances of ADR and inconsistent/irrational indication have been computed out of Unacceptable deviations in each category.

## Data Availability

The data presented in this study are available on request from the corresponding author.
